# Damage-Associated Molecular Pattern-Triggered Immunity in Plants

**DOI:** 10.3389/fpls.2019.00646

**Published:** 2019-05-22

**Authors:** Shuguo Hou, Zunyong Liu, Hexi Shen, Daoji Wu

**Affiliations:** ^1^School of Municipal and Environmental Engineering, Shandong Jianzhu University, Jinan, China; ^2^State Key Laboratory of Rice Biology, Zhejiang University, Hangzhou, China

**Keywords:** plant immunity, DAMPs, PRRs, receptor-like kinases, systemic resistance

## Abstract

As a universal process in multicellular organisms, including animals and plants, cells usually emit danger signals when suffering from attacks of microbes and herbivores, or physical damage. These signals, termed as damage-associated molecular patterns (DAMPs), mainly include cell wall or extracellular protein fragments, peptides, nucleotides, and amino acids. Once exposed on cell surfaces, DAMPs are detected by plasma membrane-localized receptors of surrounding cells to regulate immune responses against the invading organisms and promote damage repair. DAMPs may also act as long-distance mobile signals to mediate systemic wounding responses. Generation, release, and perception of DAMPs, and signaling events downstream of DAMP perception are all rigorously modulated by plants. These processes integrate together to determine intricate mechanisms of DAMP-triggered immunity in plants. In this review, we present an extensive overview on our current understanding of DAMPs in plant immune system.

## Introduction

Plants are constantly assaulted by various pathogens and insect herbivores, which seek to assimilate plant-derived nutrients for their survivals and propagations. Plants have evolved abilities to activate immune responses against invading organisms and promote wound healing. Distinct from mammals, plants lack adaptive immunity and specialized immune cells. They primarily actuate immunity by strengthening existing physical and chemical bulwarks, and activating two types of immune signaling pathways: pattern-triggered immunity (PTI) and effector-triggered immunity (ETI) (Cui et al., 2015; Yu et al., 2017). Local induction of both PTI and ETI often triggers a broad-spectrum immunity to subsequent pathogen attacks in distal tissues, a phenomenon called systemic acquired resistance (SAR) (Fu and Dong, 2013).

Pattern-triggered immunity is considered to be the first line of inducible defense in plants. It is canonically triggered through the detection of non-self microbial signatures, which are called pathogen-associated molecular patterns (PAMPs). PAMPs are often highly conserved molecules with representative characteristic of a whole class of microbes and are recognized by plasma membrane-localized pattern recognition receptors (PRRs). PRRs, such as FLAGELLIN SENSING 2 (FLS2) and EF-Tu RECEPTOR (EFR), are mainly transmembrane receptor-like kinases (RLKs) or receptor-like proteins (RLPs). Upon PAMP perception, PRRs associate or dis-associate with their partner proteins to provide a platform for downstream immune signaling, including a rapid burst of Ca^2+^ and reactive oxygen species (ROS), activation of Ca^2+^-dependent protein kinases (CDPKs) and mitogen-activated protein kinases (MAPKs), production of phytohormones, and extensive transcriptional and metabolic reprogramming (Chinchilla et al., 2007; Heese et al., 2007; Boller and Felix, 2009; Sun et al., 2013; Macho and Zipfel, 2014). ETI, in contrast, is a second layer of inducible defense typically activated by the intracellular recognition of pathogen effector molecules by plant resistance (R) gene products (Cui et al., 2015). ETI is often characterized by localized ‘hypersensitive response’ (HR), a type of programmed cell death (PCD).

**FIGURE 1:**
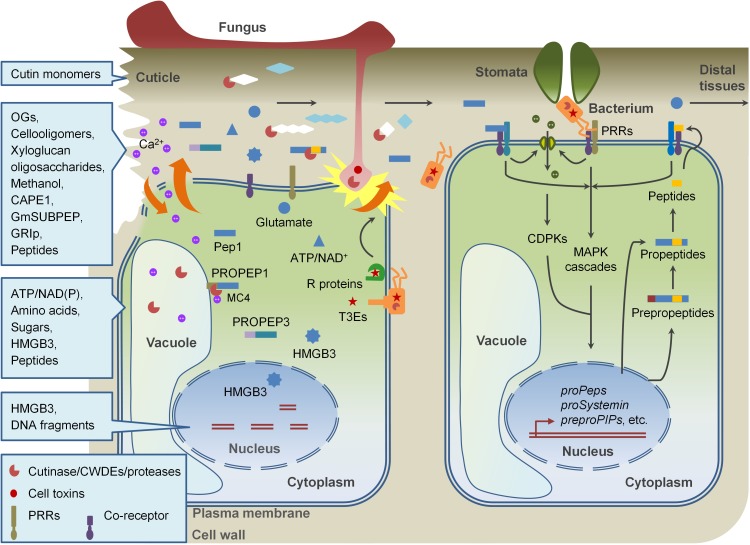
DAMP-triggered immunity in plants. Pathogen invasion disrupts plant cell wall and plasma membrane, leading to the release of DAMPs, including fragments of cell walls and apoplastic proteins, and cytoplasmic components. Perception of DAMPs as well as PAMPs by PRRs in cells surrounding of the damaged cells also promotes the production and release of new DAMPs. These DAMPs collaborating with PAMPs modulate immune responses locally and systemically.

The amplification of immune signals is very important for plants to achieve of disease resistance after when detection of PAMPs in early stages of pathogen invasion. One of the strategies is to rapidly activate immune responses by perceiving some host-derived molecules, named damage-associated molecular patterns (DAMPs), with overlapped PTI signaling components (Lotze et al., 2007; Boller and Felix, 2009). “DAMP” was originally defined in mammalian immune system in the beginning of this century and gradually referred in plant immunity (Seong and Matzinger, 2004; Lotze et al., 2007; Boller and Felix, 2009; Tanaka et al., 2014). By now, many DAMPs were identified and their roles in plant immunity are partially understood (Table 1). Similar to which in mammals, DAMPs in plants are mainly cytosolic proteins, peptide, nucleotides, and amino acids, which are released from damaged cells or secreted by intact cells undergoing pathogen invasion. In addition, some oligomeric fragments of plant cell-wall polysaccharides released when tissues are disrupted by physical injuries or attacks of pathogens and herbivores also function as DAMPs. As the case of PAMPs, DAMPs initiate PRR-mediated immune responses in local sites surrounding of wounding and pathogen invasion and regulate systemic immune signaling (Figure 1). In most cases, however, DAMPs play distinct roles from PAMPs. In this review, we summarize our current understanding of DAMPs in plant immune system, and provide an extensive overview on their molecular structures, generation, release, perception, and signaling events.

**Table 1:** Identified DAMPs in plants.

Category	DAMP	Molecular structure or epitope	Source or precursor	Receptor or signaling regulator	Plant	References
Epidermis cuticle	Cutin monomers	C16 and C18 hydroxy and epoxy fatty acids	Epidermis cuticle	Unknown	*Arabidopsis thaliana*, *Solanum lycopersicum*	Fauth et al., 1998; Buxdorf et al., 2014
Cell wall polysaccharide fragments or degrading products	OGs	Polymers of 10–15 α-1-4-linked GalAs	Cell wall pectin	WAK1 (*A. thaliana*)	*A. thaliana*, *G. max*, *N. tabacum*	Norman et al., 1999; Bellincampi et al., 2000; Ferrari et al., 2007; Brutus et al., 2010; Galletti et al., 2011
	Cellooligomers	Polymers of 2-7 β-1,4-linked glucoses	Cell wall cellulose	Unknown	*A. thaliana*	Souza et al., 2017; Johnson et al., 2018
	Xyloglucan oligosaccharides	Polymers of β-1,4-linked glucose with xylose, galactose, and fructose side chains	Cell wall hemicellulose	Unknown	*A. thaliana*, *Vitis vinifera*	Claverie et al., 2018
	Methanol	Methanol	Cell wall pectin	Unknown	*A. thaliana*, *Nicotiana tabacum*	Dixit et al., 2013; Hann et al., 2014; Tran et al., 2018
Apoplastic peptides and proteins	CAPE1	11-aa peptide	Apoplastic PR1	Unknown	*A. thaliana*, *S. lycopersicum*	Chen et al., 2014
	GmSUBPEP	12-aa peptide	Apoplastic subtilase	Unknown	*Glycine max*	Pearce et al., 2010a
	GRIp	11-aa peptide	Cytosolic GRI	PRK5	*A. thaliana*	Wrzaczek et al., 2009, 2015
	Systemin	18-aa peptide (*S. lycopersicum*)	Cytosolic prosystemin	SYR1/2 (*S. lycopersicum*)	Some *Solanaceae* species	Pearce et al., 1991; Wang et al., 2018
	HypSys	15-, 18-, or 20-aa peptides	Apoplastic or cytoplasmic preproHypSys	Unknown	Some *Solanaceae* species	Pearce et al., 2001a; Pearce and Ryan, 2003; Pearce, 2011
	Peps	23∼36-aa peptides (*A. thaliana*)	Cytosolic and vacuolar PROPEPs	PEPR1/2 (*A. thaliana*)	*A. thaliana*, *Zea mays*, *S. lycopersicum*, *Oryza sativa*	Huffaker et al., 2006; Yamaguchi et al., 2006, 2010; Krol et al., 2010; Bartels et al., 2013; Hander et al., 2019
	PIP1/2	11-aa peptides	Apoplastic preproPIP1/2	RLK7	*A. thaliana*	Hou et al., 2014
	GmPep914/890	8-aa peptide	Apoplastic or cytoplasmic GmproPep914/890	Unknown	*G. max*	Yamaguchi et al., 2011
	Zip1	17-aa peptide	Apoplastic PROZIP1	Unknown	*Z. mays*	Ziemann et al., 2018
	IDL6p	11-aa peptide	Apoplastic or cytoplasmic IDL6 precursors	HEA/HSL2	*A. thaliana*	Wang X. et al., 2017
	RALFs	∼50-aa cysteine-rich peptides	Apoplastic or cytoplasmic RALF precursors	FER (*A. thaliana*)	*A. thaliana*, *N. tabacum*, *S. lycopersicum*	Haruta et al., 2014; Stegmann et al., 2017
	PSKs	5-aa peptides	Apoplastic or cytoplasmic PSK precursors	PSKR1/2 (*A. thaliana*)	*A. thaliana*, *S. lycopersicum*	Igarashi et al., 2012; Mosher et al., 2013; Zhang et al., 2018
	HMGB3	HMGB3 protein	Cytosolic and nuclear HMGB3	Unknown	*A. thaliana*	Choi H.W. et al., 2016
	Inceptin	11-aa peptide	Chloroplastic ATP synthase γ-subunit	Unknown	*Vigna unguiculata*	Schmelz et al., 2006
Extracellular nucleotides	eATP	ATP	Cytosolic ATP	DORN1/P2K1 (*A. thaliana*)	*A. thaliana*, *N. tabacum*	Chivasa et al., 2009; Choi et al., 2014a
	eNAD(P)	NAD(P)	Cytosolic NAD(P)	LecRK-I.8	*A. thaliana*	Wang C. et al., 2017
	eDNA	DNA fragments < 700 bp in length	Cytosolic and nuclear DNA	Unknown	*Phaseolus vulgaris*, *P. lunatus*, *Pisum sativum*, *Z. mays*	Wen et al., 2009; Duran-Flores and Heil, 2014, 2018; Barbero et al., 2016
Extracellular sugars	Extracellular sugars	Sucrose, glucose, fructose, maltose	Cytosolic sugars	RGS1 (*A. thaliana*)	*A. thaliana*, *N. tabacum*, *Solanum tuberosum*	Johnson and Ryan, 1990; Solfanelli et al., 2006; Guo et al., 2011; Bolouri Moghaddam and Van den Ende, 2012; Miao et al., 2013; Li et al., 2014
Extracellular amino acids and glutathione	Proteinogenic amino acids	Glutamate, cysteine, histidine, aspartic acid	Cytosolic amino acids	GLR3.3/3.6 or others (*A. thaliana*)	*A. thaliana*, *S. lycopersicum*, *Oryza sativa*	Qi et al., 2006; Li et al., 2013; Kadotani et al., 2016; Seo et al., 2016; Toyota et al., 2018
	Glutathione	Glutathione	Cytosolic glutathione	GLR3.3/3.6 (*A. thaliana*)	*A. thaliana*	Li et al., 2013

## Cuticle and Cell Wall Components

Cuticles and plant cell walls form outermost physical obstacles encountered by pathogens. Plant cell wall is also one of the major carbon sources for necotrophic pathogens. To penetrate these barriers and assimilate nutrients from plant cells, pathogens devour their hosts by secretion of cutinolytic enzymes and cell wall degrading enzymes (CWDEs) (Kubicek et al., 2014). To counteract such savage enzymatic impacts, plants have evolved strategies to monitor the integrities of cuticles and cell walls through the perception of their degradation products, a group of DAMPs, and activate immune responses.

### Cutin Monomers

Plant cuticles are protecting films covering the epidermal cells of leaves and some other aerial plant organs. The main structural components of plant cuticles are unique polymer cutins. The insoluble polymers built mainly on esterified C16 and C18 hydroxy and epoxy fatty acids, impregnated with wax. Although plant cuticles are thought as efficient mechanical shields against pathogen invasions, they still get infection and penetration due to the secreting cutinases from pathogens (Serrano et al., 2014; Ziv et al., 2018). However, deletion of the cutinases and cutinolytic lipases in some necrotrophic fungus, such as *Botrytis cinerea*, does not hinder the pathogen to enter intact plant tissues (Reis et al., 2005). On the contrary, *Arabidopsis* leaves treated with cutinase or overexpressing pathogen cutinase gene display strong resistance toward the necrotrophic fungus *B. cinerea* (Chassot et al., 2007). In addition, mutations in various aspects of plant cutin biosynthesis genes also improve plant resistance to *B. cinerea* (Kurdyukov et al., 2006; Chassot et al., 2007; Bessire et al., 2011), suggesting that plants may able to activate immunity by monitoring of the cuticle integrity. Exogenous application of cutin monomers to plant leaves or suspension-cultured cells induces defense responses, upregulate expression of defense-related genes, and increase resistance to *B. cinerea* (Fauth et al., 1998; Buxdorf et al., 2014). Thus, it was speculated that plant cuticles could be degraded by pathogen cutinase to cutin monomers, which acting as DAMPs elicit immune responses and elevate pathogen resistance. However, the molecular mechanisms underlying cutin monomer perception and cutin monomer-regulated plant immunity remain unclear.

### Oligogalacturonic Acid

Pectin is a major component of plant cell wall matrix. It consists of a diverse group of polysaccharides. Homogalacturonan (HG), a linear homopolymer of α-(1-4)-linked D-galacturonic acid (GalA), is the most abundant pectic polysaccharide in primary cell walls. HG is easy to be targeted and hydrolyzed by pathogen or plant-derived HG-digesting enzymes, such as polygalacturonases (PGs) and pectate lyases (PLs), and OGs, the best-known plant HG-derived DAMPs, are expected to be released through the action of these enzymes (Cervone et al., 1989; Norman et al., 1999; Orozco-Cardenas and Ryan, 1999). Exogenous treatment of plants with OGs activates a wide range of defense responses, including the production of ROS (Bellincampi et al., 2000), the activation MAPKs (Galletti et al., 2011), the deposition of callose (Galletti et al., 2008), the accumulation of phytoalexins (Davis et al., 1986; Ferrari et al., 2007), and the expression of defense-related genes (Ferrari et al., 2007). As a result, it enhances the plant resistance to multiple pathogens, such as necrotrophic *B*. *cinerea*, *Pectobacterium carotovorum*, and hemibiotrophic *Pseudomonas syringae* (Ferrari et al., 2007; Benedetti et al., 2015; Davidsson et al., 2017). It was reported that the generation of elicitor-active OGs during microbial infections is promoted by plant-encoded PG-inhibiting proteins (PGIPs), which block the complete hydrolysis of HG to galacturonic acid (Benedetti et al., 2015), suggesting that OGs are generated on condition of partial inhibition of pathogen-encoded PGs. The elicitor-active OGs were predicted to be a complex of oligomers with a degree of GalA polymerization between 10 and 15 (Cote and Hahn, 1994; Federici et al., 2006; Ferrari et al., 2007). Recently, it was indicated that trimeric OGs also induced plant immune responses and enhanced pathogen resistance similar to long OGs (Davidsson et al., 2017). However, the expression of immune-related genes induced by trimers was overall lower and shorter-lasting compared to induction with long OGs. Furthermore, trimers initiate a unique down-regulation of genes associated with growth and development, leading to stunted growth of seedlings to a significantly greater extent than long OGs, implying different roles between trimeric OGs and long OGs.

OGs were suggested to be perceived by WALL-ASSOCIATED KINASE 1 (WAK1) (Brutus et al., 2010), which possesses an extracellular region containing several epidermal growth factor (EGF) repeats apart from a transmembrane domain and an intracellular cytoplasmic kinase domain. Implication of WAK1 as an OG receptor was based on protein chimeras in which the intracellular kinase domain of WAK1 was exchanged for the kinase domain of EFR (Brutus et al., 2010). These chimeras produced an EFR signaling-like response after application of OGs. WAK1 overexpression in *Arabidopsis* enhanced OG responses and increased resistance to *B. cinerea*. Furthermore, WAK1 binds *in vitro* to OGs through its N-terminal non-EGF portion of the ectodomain. However, there is still absent of genetic evidence to support that WAK1 is an OG receptor. WAK1, as well as WAK2, also bind native pectin and long pectin fragments, but the affinities are lower than OGs (Decreux and Messiaen, 2005; Kohorn et al., 2006a,b, 2014). Pectin appears to induce MAPK3 phosphorylation and regulate the expression of numerous genes involved in cell expansion through WAK2 (Kohorn et al., 2009). However, pectin does not induce immune response or enhance plant resistance to pathogens (Kohorn, 2016). This further supports that plants have evolved abilities to monitor pectin integrity to trigger immunity. It is important to determine how plants distinguish OGs from pectin by using the same family receptor candidates.

### Cellooligomers

Cellulose is the most abundant plant cell wall polysaccharide. A collection of β-1,4-glucan chains interact with each other via hydrogen bonds to form cellulose microfibrils. Breakdown of cellulose by microbial or plant glucosidases leads to the generation of cellooligomers, including cellobiose, cellotriose, and cellotetraose, and other short-chain β-1,4-linked D-glucoses. *Arabidopsis* plants exogenously treated by cellooligomers with 2-7 D-glucose repeats rapidly increase cytosolic Ca^2+^ ([Ca^2+^]_cyt_) and activate some other immune responses, such as immune-related gene expression, MAPK activation, and metabolism changes (Souza et al., 2017; Johnson et al., 2018). Cotreatments of cellobiose or cellotriose with chitooligomers or other PAMPs led to synergistic increases of immune responses (Souza et al., 2017; Johnson et al., 2018). It was thus speculated that the perception of cellulose-derived oligomers represents an additional layer of plant immunity following PAMP perception and plant cell wall breakdown during pathogen invasion.

Similar to OGs, the activities of cellooligomers for the induction of plant immune responses are also associated with their degrees of polymerization (Trouvelot et al., 2014). In contrast to cellobiose or other longer oligomers of D-glucose, cellotriose seems to exhibit strongest activity for induction of Ca^2+^ influx in *Arabidopsis*. In addition, cellotriose but not cellobiose can induce ROS production. The PAMP receptor FLS2, CHITIN ELICITOR RECEPTOR KINASE 1 (CERK1), and coreceptor BRASSINOSTEROID INSENSITIVE 1-ASSOCIATED RECEPTOR KINASE 1 (BAK1) are not required for the cellooligomer-induced calcium signaling and downstream responses in *Arabidopsis* (Souza et al., 2017; Johnson et al., 2018). THESEUS1 (THE1), a *Catharanthus roseus* RLK1-like (CrRLK1L) family RLK required for mediation of responses caused by cellulose synthesis defects does not participate in cellobiose perception (Hematy et al., 2007). Therefore, a new receptor(s) is suggested to work for specific perception of cellooligomers in *Arabidopsis*. A loss-of-function mutant of a poly(A) ribonuclease (PARN) shows impaired response to cellotriose and other cellooligomers, suggesting a post-transcriptional control of signaling component involved in cellooligomer-triggered immune activation (Johnson et al., 2018).

### Xyloglucan Oligosaccharides

Xyloglucan is the most abundant components of hemicellulose in primary cell walls of dicotyledonous plants. It is composed of a 1,4-β-glucan backbone that is further substituted with side chains containing xylose, galactose, and fucose residues (Park and Cosgrove, 2015). A recent report demonstrated that highly purified xyloglucan oligomers obtained by enzymatic extraction and purification from apple pomace can activate immune responses, including MAPK activation, callose deposition, and immune gene expression in grapevine and *Arabidopsis*, resulting in the plant resistance against necrotrophic *B. cinerea* or biotrophic *Hyaloperonospora arabidopsidis* (Claverie et al., 2018). Therefore, it is possible that xyloglucan oligosaccharides play as DAMPs, but the mechanisms remain to be investigated further.

### Methanol

Most plant-derived methanol is generated by pectin methylesterases (PMEs), which catalyze demethylesterification of the HG of cell wall pectins. Mechanical damage or herbivore attacks of plants increase the expression of PMEs and promote emission of methanol (von Dahl et al., 2006; Korner et al., 2009). Methanol is considered not only as a by-product of PME activity but also a significant signaling molecule that regulates plant resistance responses (Dorokhov et al., 2012; Komarova et al., 2014; Tran et al., 2018). It has been indicated that methanol functions as a DAMP elicitor. Methanol application for plants activates some early defense responses, including MAPK activation, [Ca^2+^]_cyt_ elevation, membrane depolarization, ethylene production, and upregulation of defense gene expression, consequently enhancing resistance against pathogens or herbivorous insects (Dixit et al., 2013; Hann et al., 2014; Tran et al., 2018). Moreover, the exposure to methanol may result in a “priming” effect on immunity of uninfected leaves or neighboring plants (Hann et al., 2014). It is unclear whether methanol signaling is also mediated by a PRR-like receptor as some other DAMPs do.

## Apoplastic Peptides and Proteins

### CAP-Derived Peptide 1

Great expression of PATHOGENESIS-RELATED (PR) proteins is a typical marker of plant immune activation in various plant species. PR-1 is a member of a broader protein family known as the cysteine-rich secretory protein, antigen 5, and pathogenesis-related-1 (CAP) protein superfamily. The protein is antimicrobial by sequestering sterols from the membranes of microbes. It is thus more effective against sterol auxotrophs, which must obtain sterols from the environment (Gamir et al., 2017). Overexpression of PR-1 in plants results in increased resistance to fungi, oomycetes, and bacteria (Sela-Buurlage et al., 1993; Niderman et al., 1995; Selitrennikoff, 2001; Sinha et al., 2014; Breen et al., 2017).

A PR1-derived peptide, named CAP-derived peptide (CAPE) 1, was recently isolated from apoplastic fluids of tomato leaves. CAPE 1 is composed of the last 11 amino acids (aa) from the C-terminus of tomato PR-1 (Chen et al., 2014). Conserved CNYx motif positioned N-terminal to the CAPE peptide in PR-1 was confirmed to be required for the PR1 cleavage and CAPE1 release (Chen et al., 2014). CAPE1 is greatly produced in tomato leaves after damaged or pretreated with methyl jasmonate (MeJA), suggesting that an unknown protease(s) required for PR-1 cleavage and CAPE1 release is activated by wound and MeJA. Tomatoes pretreated with CAPE1 lead to the expression of multiple defense-related genes, the induction of salicylic acid (SA) and JA biosynthesis, and the enhancement of the resistance to herbivore *Spodoptera litura* larvae and bacterial *P. syringae* pv. tomato (*Pst*) strain DC3000. Application of a synthetic peptide predicted to be a CAPE derived from *Arabidopsis* PR-1 also activate the resistance against *Pst* DC3000 infection (Chen et al., 2014), implying that CAPE releases from PR proteins represent a conserved mechanism of plant immune regulation. However, CAPE1 cannot upregulate expression of *WRKY53*, which is highly upregulated in response to the application of the PAMP flg22 or some other DAMPs (Chen et al., 2014). Thus, CAPE1 may not induce the canonical PTI signaling.

### *Glycine max* SUBTILASE PEPTIDE

Subtilisin-like proteases (SBTs) constitute a large family of extracellular plant serine proteases (Schaller et al., 2018). Some of them are involved in plant resistance to pathogens (Figueiredo et al., 2018; Hou et al., 2018). For example, tomato subtilases, P69B and P69C, are induced by pathogen attack and SA application and secreted into plant extracellular matrix to protect plants against pathogen infection (Tornero et al., 1996a,b; Jorda and Vera, 2000). AtSBT3.3, an ortholog of tomato P69C in *Arabidopsis*, positively regulates plant resistance to the bacterial pathogen *P. syringae* and the oomycete pathogen *H. Arabidopsidis* (Ramirez et al., 2013). It was reported that a 12-aa peptide elicitor, named Soybean (*Glycine max*) SUBTILASE PEPTIDE (GmSUBPEP), was processed from a unique region of a subtilase in legume plants (Pearce et al., 2010a). Exogenous application of synthetic GmSUBPEP on soybean leaves induced expression of defense-related genes, illustrating that the peptide plays as a DAMP. How GmSUBPEP is processed and perceived to activate resistance responses in legume plants remains to be determined in the future.

### GRIM REAPER Peptide (GRIp)

GRI is an *Arabidopsis* ortholog of the tobacco flower-specific Stig1. It was originally identified for the function in ozone response (Wrzaczek et al., 2009). A gain-of-function mutant of *GRI*, expressing more GRI-derived peptide, is more sensitive to ozone. Moreover, this mutant not only shows the increasing resistance to a virulent bacterial pathogen *Pst* DC3000, but also speeds up the induction of cell death to avirulent pathogen *Pst* DC3000 *avrRpt2* (Wrzaczek et al., 2009). A subsequent study indicated that GRI protein is localized in extracellular spaces of *Arabidopsis* leaves, where it appears to be processed by METACASPASE9 (MC9) with a release of an N-terminal 11-aa peptide, GRIp. The released GRIp is perceived by the plasma membrane-localized, atypical LRR-RLK POLLEN-SPECIFIC RECEPTOR-LIKE KINASE 5 (PRK5), and induces SA and extracellular superoxide-dependent cell death (Wrzaczek et al., 2015). However, it is largely unclear how GRIp perception leads to the resistance to *Pst* DC3000.

### Systemin and HypSys

Systemin is the first reported plant peptide signal, which exists in most species of the *Solanaceae* family including tomato and potato but excluding tobacco (Pearce et al., 1991; McGurl et al., 1992). It is an 18-aa peptide and is synthesized as a 200-aa precursor protein named prosystemin. Prosystemin does not carry an N-terminal secretion signal sequence. It massively accumulates in the cytosol of tomato cells in response to wounding, herbivore attack or treatment with MeJA (Narvaez-Vasquez and Ryan, 2004). Exogenous application of systemin activates defense-related responses, including the production of protease inhibitors, the increase of ethylene and MeJA biosynthesis, the induction of an oxidative burst, and the enhancement of tomato resistance against insect herbivory (Schilmiller and Howe, 2005). Overexpression of prosystemin causes the constitutive production of protease inhibitors in plants even in the absence of wounding (Ryan and Pearce, 1998; Pearce, 2011). Thus, systemin is likely to be released through a combination of unconventional protein secretion and plant tissue injury. More recently, it was shown that the RLK SYSTEMIN RECEPTOR 1 (SYR1) acts as a systemin receptor, and both SYR1 and its homologous SYR2 are required for systemin perception in species of the *Solanoideae* subfamily (Wang et al., 2018). Prosystemin appears to be synthesized within the vascular bundles where wounding would release systemin in the vicinity of the phloem for the transport to other parts of the plant. As the name implies, systemin was suggested to be a mobile signal for the induction of systemic wounding signaling in some initial researches. However, it was recently indicated that systemic responses caused by mechanical damage are irrespective of the presence or absence of SYR1 and SYR2 in the tomato (Wang et al., 2018), implying that other long-distance signals, rather than systemin, are essential for systemic wound responses. The hydroxyproline-rich systemins (HypSys), a class of systemin-like peptides, are also produced and induce the synthesis of defensive proteinase inhibitor proteins in some *Solanaceous* plants like tomato and tobacco (Pearce et al., 2001a; Pearce and Ryan, 2003; Pearce, 2011). These peptides are hydroxylated, glycosylated and constitutively presented in apoplastic spaces of plants as they are originated from larger pre-proprotein precursors with signal peptides for secretion. However, HypSys do not share sequence homology with systemin, although they trigger physiological responses same as tomato systemin.

### Plant Elicitor Peptides (Peps)

Plant elicitor peptide 1 (Pep1) is the first peptide elicitor identified in *Arabidopsis thaliana*. It is 23-aa long and derived from the carboxyl end of an approximately 100-aa long precursor protein, PROPEP1 (Huffaker et al., 2006; Yamaguchi et al., 2006). *At*Pep1 promotes plant resistance to various pathogens, including bacterial *Pst* DC3000, fungal *B. cinerea* and *Phytophthora infestans* (Huffaker et al., 2006; Yamaguchi et al., 2010; Liu et al., 2013). *Arabidopsis* encodes eight PROPEP paralogs (PROPEP1-PROPEP8) that harbor conserved Pep epitopes in their C-termini. Similar to prosystemin, PROPEPs lack an N-terminal signal sequence to enter the canonical secretory pathway (Huffaker et al., 2006; Bartels et al., 2013). Individual PROPEPs have been shown to localize to the cytosol or to be associated with the tonoplast (Bartels et al., 2013), but it is perceived outside of cells, contributing to the assumption that Peps are released into the apoplast during cell damage. A recent paper revealed that PROPEP1 is sequestered at the vacuolar membrane in the absence of damage, but it is processed by METACASPASE4 (MC4) and released only in damaged cells (Hander et al., 2019). Once cells damaged, MC4 is activated by an prolonged increase of the [Ca^2+^]_cyt_ and consequently cleaves and releases the active elicitor Pep1 from PROPEP1. Pep3 seems to be greatly released out of cells in *Arabidopsis* seedlings challenged with virulent and avirulent bacterial pathogen *P. syringae* (Yamada et al., 2016b), implying that pathogen invasion might promote Pep maturation and extracellular release. Importantly, unprocessed PROPEP may also be released when cells are not totally disrupted as the propeptide PROPEP3 is detectable in the extracellular space of *Arabidopsis* seedlings upon treatment with Pep2 or isoxaben (ISX), a herbicide that blocks cellulose biosynthesis (Yamada et al., 2016b; Engelsdorf et al., 2018). Thus, Peps might be released out of cells via both unconventional secretion routes and cell damage.

Two homologous LRR-RLKs, PEP RECEPTOR 1 (PEPR1) and PEPR2, were identified as the receptors of Peps in *Arabidopsis* (Yamaguchi et al., 2006, 2010; Krol et al., 2010). PEPR1 is able to recognize all eight Peps, but PEPR2 detects only Pep1 and Pep2. Structural and biochemical analyses reveal that AtPep1 binds the PEPR1-LRR domain, triggers heterodimerization between PEPR1 and its coreceptor BAK1, and BAK1-dependent PEPR activation (Schulze et al., 2010; Tang et al., 2015). Once perceived, Pep1 activates various PTI responses (Huffaker et al., 2006; Ranf et al., 2011; Bartels et al., 2013; Liu et al., 2013; Ma et al., 2013; Hou et al., 2014). Pep-PEPR1 also contributes to co-activation of SA, JA, and ethylene-mediated immune pathways (Ma et al., 2012; Flury et al., 2013; Tintor et al., 2013; Ross et al., 2014). In addition, Pep-PEPR triggers systemic immunity as well since local AtPep2 application co-activates of SA and JA-mediated responses and enhances resistance to *Pst* DC3000 in distal leaves, and PEPR1 and PEPR2 are required for *Pst* DC3000, *Pst* DC3000 avrRpm1 and flg22 induced systemic expression *PR* genes or resistance to *Pst* DC3000 (Ross et al., 2014). It was shown that PROPEP2 and PROPEP3 are expressed in local, not in systemic leaves upon *Pst* DC3000 infection. PROPEP3 protein is also only detected in local but not distal leaves, excluding a possibility that Pep peptides are long-distance signals for the systemic immune activation (Ross et al., 2014).

The function of Pep family as endogenous regulators of innate immunity is conserved across diverse plant species. As an ortholog of AtPep1, maize ZmPep1 was demonstrated to promote accumulation of transcripts and metabolites associated with pathogen defense to enhance resistance against the fungal pathogens *Cochliobolus heterostrophus* and *Colletotrichum graminicola* (Huffaker et al., 2011). ZmPep1 was also proved to be perceived by ZmPEPR1, a maize ortholog of AtPEPR1 (Lori et al., 2015). In contrast, ZmPep3 as well as many Pep orthologs from other species, such as rice, soybean, and eggplant, were shown to facilitate the emission of herbivory-associated volatiles to improve the resistance to the herbivore *Spodoptera exigua* (Huffaker et al., 2013).

### GmPep914/GmPep890

GmPep914 and GmPep890 are two 8-aa long homologous peptides. Like GmSubPep, they were isolated from leaf extracts of soybean and identified as alkalinization factors of suspension-cultured cells (Yamaguchi et al., 2011). Both peptides are processed from the C-terminus of their precursor proteins, GmPROPEP914 and GmPROPEP890, by an unknown protease(s). They can induce the expression of their precursor genes and defense genes involved in pathogen defense. Similar to prosystemins and PROPEPs, GmPROPEP914 and GmPROPEP890 have no obvious N-terminal signal sequence for secretion. It is unclear how the two peptides are released into apoplastic and perceived by cell surface-localized receptor.

### *Zea mays* Immune Signaling Peptide 1 (Zip1)

*Zea mays* immune signaling peptide 1 (Zip1) is a 17-aa peptide which was isolated from apoplastic fluids of SA-pretreated leaves in maize. The peptide is processed from its propeptide precursor, PROZIP1, by papain-like cysteine proteases (PL), CP1 and CP2, -mediated protein cleavage. Zip1 treatment strongly elicits SA accumulation, induces highly overlapping transcriptional changes associated with SA-responsive genes, and confers maize an increased susceptibility toward the necrotrophic pathogen *B. cinerea* but a reduced infection of the biotrophic fungus *Ustilago maydis*. However, different from most DAMPs in plants, Zip1 is unable to induce rapid ROS production and MAPK phosphorylation, suggesting that Zip1 lacks activities to activate common PTI signaling in maize. Zip1-treated leaves of maize displayed strong induction of apoplastic PLCP activity comparable to the treatment by SA infiltration. Therefore, a positive feedback loop for amplification of SA signaling and ultimate immune activation was proposed, in which SA burst in plants upon pathogen infection induces PLCPs-dependent release of active Zip1 from PROZIP1, Zip1 in turn induces the activity of the PLCP proteases and SA production. However, PROZIP1 transcripts are not induced by Zip1 or SA (Ziemann et al., 2018).

### PIP1

PAMP-induced peptides (PIPs) represent another family of *Arabidopsis* peptide elicitors. The group peptides were supposed to be processed from conserved carboxyl-termini of a family of preproprotein precursors (pre-proPIPs) which were identified as MAMP-upregulated gene products. Sequences homologous to AtPIPs are found in genomes of numerous monocot and eudicot species. *Arabidopsis* harbors 11 members of preproPIPs, three (pre-proPIP1, pre-proPIP2, and pre-proPIP3) of which are induced by MAMPs, SA, or pathogen infection. AtPIP1 and AtPIP2, peptides corresponding to conserved carboxyl-termini of preproPIP1 and preproPIP2, are able to activate PTI-like responses and amplify flg22-induced defenses, and enhance *Arabidopsis* resistance against *Pst* DC3000 and *Fusarium oxysporum*. As an XI subgroup of LRR-RK, RLK7, was confirmed to be a receptor of PIP1. Different from precursor proteins (systemin, Peps, and Zip1), PIP1 can be automatically secreted into extracellular spaces in a signal peptide-dependent manner and processed extracellularly into mature peptides by an unknown protease(s) (Hou et al., 2014).

### IDA-Like Peptides

Like *prePROPIP1*, *INFLORESCENCE DEFICIENT IN ABSCISSION* (*IDA*) and *IDA-LIKE 6* (*IDL6*) were identified as potential peptide precursor genes which are upregulated by PAMPs (Hou et al., 2014; Wang X. et al., 2017). IDL6 is one of the seven homologous proteins of IDA, the mature peptide of which was confirmed to control floral organ abscission and lateral root emergence upon perception by its receptor LRR-RK HAESA (HAE) and HAESA-LIKE2 (HSL2) (Butenko et al., 2003; Stenvik et al., 2008; Kumpf et al., 2013). IDA is mainly expressed in flower abscission zones and lateral root primordia, while IDL6 is prominently produced in *Arabidopsis* leaves. The expression of IDL6 is significantly upregulated by attacks of the bacterial *Pst* DC3000. IDL6 promotes HAE/HSL2-mediated susceptibility of *Arabidopsis* to *Pst* DC3000 by manipulating pectin digestion and inhibiting SA signaling (Wang X. et al., 2017). HAE is also expressed in abscission zones of *Arabidopsis* leaves upon infection with *Pst* DC3000. *Pst* DC3000 also triggers HAE/HSL2-dependent cauline leaf abscission (Patharkar et al., 2017). Therefore, an IDA family peptide might be essential for *Pst* DC3000-induced leaf abscission process. Furthermore, the pathogen-triggered leaf abscission was proposed to be an active defense response which is exploited by plants to prevent spread of the infection to the rest of the plant.

### RALFs

RAPID ALKALINIZATION FACTORs (RALFs) are a family of cysteine-rich peptides, which are generated from pre-proproteins (Pearce et al., 2001b, 2010b). The RALF peptides contain four conserved cysteines, which are thought to form two disulfide bridges and are important for protein conformation. RALF family is wildly present across the land plants including the eudicots and the monocots. *Arabidopsis* genome contains a big RALF family with 39 family members. Several of them, including RALF1 and RALF23, were confirmed to be perceived by the receptor CrRLK1L RLK FERONIA (FER) (Haruta et al., 2014; Stegmann et al., 2017).

RALFs have been shown to positively and negatively regulate plant immunity. They were thus suggested to functionally resemble interleukins, a group of cytokines involved in the regulation of immune and inflammatory responses in mammals (Banchereau et al., 2012; Gust et al., 2017). In *Arabidopsis*, RALF23 negatively regulate PAMP responses and resistance to *Pst* DC3000. The inhibition of PTI is mediated by its receptor FER, which was suggested to act as a scaffold to promote the formation of active heteromeric PRR signaling complexes between the PAMP receptor FLS2 or EFR and their co-receptor BAK1 (Stegmann et al., 2017). RALF23 peptide treatment suppresses ligand-induced heteromerization between FLS2 or EFR and BAK1. The peptide is cleaved at a RRXL site by the serine protease SITE-1 PROTEASE (S1P), leading to the generation of its mature peptide which, is essential for its negative regulation of plant immunity (Srivastava et al., 2009; Stegmann et al., 2017). On the contrary, RALF17 is not cleaved by S1P and was shown to induce PTI response and promote elf18-induced PTI activation, thus contributing to *Arabidopsis* resistance to *Pst* DC3000 (Stegmann et al., 2017). Interestingly, the RALF17 function is also FER dependent. However, it is undisclosed how the two homolog peptides play reverse functions through the same receptor.

Apart from fine-tuning PTI, RALF23 perception by FER also inhibits JA signaling and thus promotes SA signaling. SA and JA function as two major immune-related hormones in plants. SA-mediated resistance is usually effective against biotrophs and hemi-biotrophs, while JA-mediated responses are predominantly against necrotrophs and herbivorous insects. Both signaling pathways are often mutually antagonistic. The transcription factor MYC2 as well as its close paralogs, MYC3 and MYC4, are key regulators of JA signaling pathway in *Arabidopsis* (Kazan and Manners, 2013). FER was found to inhibit JA signaling by phosphorylating and destabilizing MYC2. RALF23 can act through FER to stabilize MYC2 and enhance MYC2-mediated JA signaling, thus negatively contributing to plant immunity to biotrophs (Guo et al., 2018).

### Phytosulfokine

Phytosulfokines (PSKs) are sulfated tyrosines containing pentapeptides, which are processed from approximately 80-aa pre-propeptides by post-translational sulfation and proteolytic cleavage (Matsubayashi and Sakagami, 1996; Yang et al., 1999, 2001; Srivastava et al., 2008; Komori et al., 2009). They are ubiquitously present in higher plants. In *Arabidopsis*, two plasma membrane-localized LRR-RLKs PSK RECEPTOR 1 (PSKR1) and PSKR2 are PSK receptors (Matsubayashi et al., 2006; Amano et al., 2007). Structural analyses revealed that PSK interacts mainly with the island domain embedded in PSKR extracellular LRR repeats. PSK binding promotes PSKR heterodimerization with SOMATIC EMBRYOGENESIS RECEPTOR KINASEs (SERKs), resulting in the activation of PSKR (Wang et al., 2015). In *Arabidopsis*, several PSKs were shown to be upregulated by the PAMP flg22 treatment (Hou et al., 2014). After perception by PSKR1/2 (mainly PSKR1), PSK attenuates PTI responses (Igarashi et al., 2012), decreases the resistance to the hemibiotrophic bacterial pathogen *P. syringae*, but enhances resistance against the necrotrophic fungal pathogen *Alternaria brassicicola* (Mosher et al., 2013). The PSK mediates immune regulation by suppressing the SA-mediated defense signaling but enhancing the JA-mediated defense signaling (Mosher et al., 2013). The PSK function also works in other plants. In *Zinnia elegans*, PSK treatment reduces the transcription of *PR* gene *ZePR1* and the protease inhibitor genes *ZePI1* and *ZePI2*, whereas inhibition of PSK synthesis results in increased transcription of these genes (Motose et al., 2009). A recent report demonstrated that PSK acts as a DAMP and contributes to the immunity to necrotrophic fungal pathogen *B. cinerea* in tomato plants (Zhang et al., 2018). Transcription of genes encoding several PSK precursors as well as the PSK sulfation processing gene *TYROSYLPROTE SULFOTRANSFERASE* (*TPST*) was significantly upregulated by *B. cinerea* inoculation. However, unlike *Arabidopsis*, the PSK-mediated resistance to *B. cinerea* in tomatoes does rely on auxin-dependent immune responses but not on SA and JA signaling pathways. In mechanism, the PSK recognition by tomato PSKR1 elevated [Ca^2+^]_cyt_ promote the binding between calmodulins (CaMs) and the auxin biosynthetic YUCCAs (YUCs) and facilitates the auxin accumulation, leading to the activation of auxin-mediated immunity.

### High Mobility Group Box-3 (HMGB3)

HMGBs are a family of highly conserved nuclear proteins expressed in most eukaryotic cells. They participate in the organization, stabilization and repair of genomic DNA, and transcription regulation. In mammals, HMGB1 is the first biomolecule defined as a DAMP, which is released extracellularly in case of cell death or tissue injury to activate inflammatory and immune responses (Lotze and Tracey, 2005). In *Arabidopsis*, at least 15 genes encode HMG-box domain-containing proteins. AtHMGB3 localizes in the nucleus and cytoplasm and has been shown to be released into the extracellular spaces during the infection of the necrotrophic pathogen *B. cinerea*. Exogenous treatment with AtHMGB3 protein induces PTI responses, including MAPK activation, defense-related gene expression, callose deposition, and activates JA/ethylene-associated defenses to *B. cinerea* (Choi H.W. et al., 2016). Thus, AtHMGB3 was suggested to be a DAMP, which mainly protect plants against necrotrophs and insects, rather than against biotrophs and hemi-biotrophs. Like its human counterpart, AtHMGB3 binds to SA, resulting in the inhibition of its DAMP activity (Choi H.W. et al., 2016). Infection with a biotrophic pathogen leads to an increase in SA levels, the elevated SA might antagonize the activation of JA-mediated defenses in part by suppressing AtHMGB3’s DAMP activity, thus directs plants to the SA-mediated resistance against biotrophic and hemi-biotrophic pathogens.

### Inceptin

When cowpea (*Vigna unguiculata*) leaves are consumed by armyworm (*Spodoptera frugiperda*) larvae, a proteolysis fragment of the cowpea chloroplastic ATP synthase γ-subunit (also named cATPC protein), termed inceptin, was produced in gut of the insect (Schmelz et al., 2006). Inceptin is a disulfide-bridged peptide containing 11 amino acids. Exogenous treatment of the peptide promotes the production of immune-related hormones, ethylene, SA and JA, and defense metabolite cinnamic acid in cowpea, upregulates transcription of cowpea protease inhibitor, and enhances cowpea resistance to herbivore attacks. Sequence alignments of cATPC proteins from multiple plant species demonstrate a high degree of conservation in the amino acid sequence related to the predicted inceptin peptides. However, inceptins are active elicitors of defense responses only in one clade of the *Fabaceae* (Schmelz et al., 2007), suggesting that inceptin perception is a recent evolutionary event in plants.

## Extracellular Nucleotides

### Extracellular Adenosine 5-Triphosphate (ATP)

ATP is a universal energy source to drive many biochemical reactions in cells. Intracellular ATP can be released into the extracellular matrix, where it is referred to as extracellular ATP (eATP), either in response to environmental stimuli or physical cell injury. In animals, eATP is considered to be a DAMP, which is perceived by two types of receptors, ligand-gated ion channel P2X and G protein-coupled P2Y, and regulate a variety of responses, such as neurotransmission, inflammation, and cell death (Khakh and Burnstock, 2009). Wounding, pathogen infection, or insect infestation also cause plants release of high level of ATP (approximate 40 μM) into the extracellular matrix, where it is recognized as a DAMP to initiate plant resistance responses (Chivasa et al., 2009; Choi et al., 2014a,b; Medina-Castellanos et al., 2014). Exogenous application of ATP triggers signaling pathways similar to the PAMP-induced plant defense responses, including elevation of [Ca^2+^]_cyt_, production of nitric oxide (NO) and ROS, and MAPK phosphorylation. Gene transcriptome analysis indicated that eATP induces the expression of a large proportion of genes overlapped with those responding to wound and JA (Choi et al., 2014a; Tripathi et al., 2018; Jewell et al., 2019). eATP treatment enhances the interaction between JA receptor CORONATINE 1 (COI1) and JASMONATE ZIM-DOMAIN 1 (JAZ1), leading to a proteasome-dependent JAZ1 degradation (Tripathi et al., 2018). Therefore, eATP may promote JA signaling by eliminating JAZ1 inhibition on JA signaling. Moreover, eATP also regulates gene expression through pathways independent of COI1 but reliant on MYC transcription factors and CALMODULIN-BINDING TRANSCRIPTION ACTIVATOR 3 (CAMTA3) (Jewell et al., 2019).

In *Arabidopsis*, an L-type lectin receptor kinase, DOESN OT RESPOND TO NUCLEOTIDES 1 (DORN1) (also known as P2K1), has been implicated in eATP sensing and signaling (Choi et al., 2014a). The extracellular legume L-type lectin domain of the DORN1/P2K1 binds ATP with high affinity, but the exact ATP binding site is still unclear. In addition to ATP, some other nucleotides, such as ADP, ATPγS, ADPβS, GTP, UTP, and CTP, are also active in stimulation of calcium influx, but AMP and adenosine have no detectable activities (Tanaka et al., 2010). DORN1/P2K1 is required for the perception of the most nucleotides with a preference for purine nucleotides over pyrimidine nucleotides (Choi et al., 2014a).

### Extracellular Nicotinamide Adenine Dinucleotide (NAD) and NAD Phosphate (NADP)

NAD and NADP are universal coenzymes, which participate in both metabolic reactions and intracellular signaling. In *Arabidopsis*, exogenous NAD(P)H and NAD(P)^+^ were shown to activate immune responses, including SA accumulation, *PR* gene expression, and enhance plant resistance to the *Psm* ES4326 (Wang C. et al., 2017). Transcriptome analysis revealed that NAD upregulates large number genes involved in PTI and SA signaling pathways but suppresses expression of several genes in JA/ET pathway, pointing that NAD mainly triggers plant resistance against biotrophic and hemibiotrophic pathogens. Importantly, pathogen infection and wounding commonly cause intercellular NAD(P) leakage into the extracellular space at concentrations sufficient to induce these immune responses (Wang C. et al., 2017). Thus, eNAD(P) was suggested to act as a DAMP in plants. Furthermore, eNAD(P) was found to play a critical role in the regulation of SAR because eNAD(P) depletion *in planta* through transgenic expression of the human NAD(P)-metabolizing ectoenzyme CD38 compromises SAR induction by local infection with the avirulent bacterial pathogen *Pst* DC3000 avrRpt2. However, exogenous application of NAD(P) is unable to induce SAR, suggesting that eNAD(P) alone is not sufficient for activation of SAR (Zhang and Mou, 2012). Mutations in LecRK-I.8, a DORN1/P2K1 homologous lectin-receptor kinase, partially compromise NAD-induced immune responses and disease resistance. LecRK-I.8 has low affinity to NAD, but not affinity to ATP and NADP^+^, implying that LecRK-I.8 plays as a putative eNAD receptor in *Arabidopsis* (Wang C. et al., 2017).

### DNA Fragments

DNA fragmentation and the release of the fragmented DNA occur during cell death due to pathogen infection or other stresses in plants and animals (Ryerson and Heath, 1996; Kuthanova et al., 2008). In animals, self extranuclear DNA (eDNA) released from dead cells is typical DAMP and it is perceived by TOLL-LIKE RECEPTOR 9 (TLR9) and other sensors of neighboring cells to activate innate immune responses (Gallucci and Maffei, 2017). Emerging evidences suggested that self eDNA fragments (below a size of 700 bp) also play as DAMPs to activate various PTI responses, such as membrane depolarization, Ca^2+^ influx, ROS generation, and MAPK activation, and increase resistance to pathogen infections in plants (Wen et al., 2009; Duran-Flores and Heil, 2014, 2018; Barbero et al., 2016). However, non-self DNA obtained from different plant species exhibits significantly lower or no detectable activities for immune activation (Duran-Flores and Heil, 2018), pointing a species-specific discrimination of self eDNA from non-self eDNA in plants. Activation of typical PTI responses by self eDNA also suggests the sensing of self eDNA depends on an PRR-like receptor(s). However, no such receptor has been definitively identified yet. The eATP receptor DORN1/P2K1 and the eNAD receptor LecRK-I.8 belong to L-type lectin receptor kinases, which are thus suggested to specifically recognize nucleotides. Therefore, it is conceivable to argue that the L-type lectin receptor kinase might be the putative receptor of plant eDNA. Further studies are required to elucidate the nature of plant eDNA receptors.

## Extracellular Sugars

Sugars represent a group of molecules that are well-known for their roles in energy metabolism. Some bacterial and fungal pathogens assimilate the host plant-released sugars to survive and propagate in plant apoplastic spaces. To acquire abundant sugars, pathogens causes the leakage of intercellular sugars to the extracellular spaces through disintegrating plasma membrane or promoting sugar efflux into the apoplast by manipulating host plant plasma membrane-localized sugar transporters. For example, *Xanthomonas* bacteria deliver transcription activator-like (TAL) effector proteins into leaf cells to induce expression of genes encoding sugar transporters to cause plant to release more sucrose (Cohn et al., 2014; Cox et al., 2017). As the opposite site, the plant also controls sugar transporter activity, to redistribute the sugars away from the infection niche, removing the energy source to limit pathogen proliferation. For instance, the recognition of the PAMP flg22 by its receptor FLS2 and coreceptor BAK1 in *Arabidopsis* leads to the phosphorylation of the plasma membrane SUGAR TRANSPORTER 13 (STP13) to promote its activity for monosaccharide uptake from the apoplast (Yamada et al., 2016a).

In addition to their fundamental roles as carbon and energy sources, extracellular sugars also act as signaling molecules to regulate plant resistance responses. Exogenous application of sucrose, glucose, or sucrose isomers activate MAPKs and induce the expression of several *PR* genes (Bolouri Moghaddam and Van den Ende, 2012). Besides, sucrose, glucose, fructose, and maltose also specifically stimulate the accumulation of JA and antimicrobial agent anthocyanins, isoflavonoids, and glucosinolates in *Arabidopsis* (Solfanelli et al., 2006; Guo et al., 2011; Miao et al., 2013; Li et al., 2014), and enhance the expression of wound-inducible proteinase inhibitor genes in tobacco and potato tissues (Johnson and Ryan, 1990). Therefore, extracellular sugars may be recognized as DAMPs, triggering the plant defense signaling. Mounting evidence shows that extracellular sugars are sensed by REGULATOR OF G-PROTEIN SIGNALING 1 (RGS1), a seven-transmembrane receptor coupled with heterotrimeric G proteins (Johnston et al., 2007). It was indicated that glucose induces a WITH NO LYSINE 8 (WNK8)-dependent phosphorylation of RGS1 and a consequent endocytosis of RGS1 (Urano et al., 2012). RGS1 endocytosis leads to the physical uncoupling of RGS1 from the *Arabidopsis* Gα subunit, GPA1, and thus lifts the suppression on G-protein-mediated signaling. However, a direct relationship between RGS1 endocytosis and glucose-induced immune signaling has not yet built.

## Extracellular Amino Acids and Glutathione

### Amino Acids

Earlier evidences for endogenous amino acids acting as extracellular signals involved in plant signaling regulation mainly based on the discovery of ionotropic glutamate receptors (iGluRs)-like genes (*GLRs*) in plants (Lam et al., 1998). In mammals, iGluRs as ligand-gated ion channels are gated by glutamate which is known as key neurotransmitters in the central nervous system. The plant GLRs are predicted to have similar structures as their mammalian homologs, with an N-terminal ligand-binding domain, a transmembrane domain that includes a pore region, and a C-terminal domain. In contrast to the ligand specificity of the iGluRs, the GLR receptors in plants are gated by a broad spectrum of amino acids. At least 6 of the 20 proteinogenic amino acids, including glutamic acid, glycine, cysteine, serine, alanine, and asparagine, can serve as GLR agonists in *Arabidopsis* (Qi et al., 2006; Stephens et al., 2008; Li et al., 2013; Tapken et al., 2013). These amino acids trigger the membrane depolarization and the [Ca^2+^]_cyt_ elevation in a GLR3.3-dependent manner (Stephens et al., 2008). Among these, cysteine triggers defense responses and enhance plant resistance to pathogens through GLR3.3 in *Arabidopsis*. Aspartic acid cannot trigger [Ca^2+^]_cyt_ elevation but increase the GLR3.3-independent resistance to *Pst* DC3000. Loss-of-function mutations of the *Arabidopsis GLR3.3* increased susceptibility to *P. syringae* and *H. Arabidopsidis* (Li et al., 2013; Manzoor et al., 2013). In addition, histidine was also reported to induce resistance in *Arabidopsis* and tomato against the soil-borne bacterial pathogens *Ralstonia solanacearum* and *B. cinerea*, through activation of ethylene signaling pathway (Seo et al., 2016). Glutamic acid elevates resistance to the blast fungus *Magnaporthe oryzae* in rice (Kadotani et al., 2016) and the necrotrophic fungus *Alternaria alternata* in tomato (Yang et al., 2017), but it seems not to promote pathogen resistance in *Arabidopsis* (Li et al., 2013).

After sensing local herbivore attacks, plants transmit this information throughout the whole plants to rapidly enhance defense to insect herbivores in distal undamaged parts. The systemic defense was previously marked by the expression of wound-responsive marker genes and the accumulation of JA and JA-isoleucine (JA-Ile). Recent studies also detected long-distance electrical and calcium signals convert local wounding signaling (Mousavi et al., 2013; Choi W.G. et al., 2016; Toyota et al., 2018). These wound-induced systemic calcium response, as well as defense gene expression and JA and JA-Ile accumulation, are eliminated in *atglr3.3*/*atglr3.6* double mutants (Toyota et al., 2018). The AtGLR3.3/AtGLR3.6-dependent systemic calcium signaling can be mimicked by local application of glutamate instead of other amino acids (Toyota et al., 2018). Importantly, apoplastic glutamate concentration is increased by wounding, and this signal moves along the vasculature. Thus, glutamate was proposed as a DAMP, which is released into apoplastic spaces upon herbivore and mechanical damage. Released glutamate could travel long distances and activate GLR3 ion channels in the plasma membrane of cells that line the vasculature, where leading to JA accumulation and conveying host resistance to herbivory insects.

### Glutathione

Reduced tripeptide glutathione (GSH) is the most abundant short-chain peptide in cells representing the major intracellular pool of endogenous non-protein thiols and protecting cell membranes against oxidative stress. In plants, pathogen infection increases the accumulation of GSH in plant cells and causes releases of the cytosolic GSH through oligopeptide transporters or disintegrating plasma membrane (Vanacker et al., 2000; Parisy et al., 2007). GSH treatment activates typical immune responses and suppresses pathogen propagation through the AtGLR3.3-dependent pathway (Wingate et al., 1988; Li et al., 2013), implying that extracellular GSH represents a kind of DAMP functionally resembling amino acids.

## Concluding Remarks

Plants activate the plasma membrane localized PRR-mediated immune responses through the detection of extracellular danger signals, including pathogen-derived PAMPs and plant-derived DAMPs. Immune activation is energy-consumed process, which is disadvantageous to plant normal growth. To reduce losses, plant endogenous molecules which potentially play as DAMPs are produced and exposed to the surfaces of the plasma membrane and detected by PRRs when plants are suffering from pathogens invasion. The DAMPs not only contain molecules which are released on conditions of cell damage, but also include extracellular and intercellular molecules which are greatly produced, secreted, or released from their precursors without cell injury when pathogen infection. A great many researches revealed that DAMP-triggered immunity shares overlapped signaling components and functional mechanisms with PTI pathways. For example, some peptide-type DAMPs are perceived by LRR-RLKs which are close to the PAMP flagellin receptor FLS2 and EF-Tu receptor EFR. Like flagellin and EF-Tu, the perception and downstream signaling of these DAMPs usually involve BAK1, MAPK cascades, and some other PTI components. DAMPs also reprogram plant transcriptome and metabolome resembling PAMPs. In these respects, DAMPs function as PTI amplifiers. DAMP signaling also is essential for the compensation of MAMP signaling when MAMP-triggered defenses are compromised. In some cases, DAMPs play different roles from MAMPs. For example, PSKs and RALF23 negatively regulate PTI signaling, while CAPE1 and Zip1 act in regulation of SA signaling or other immune-related branches but not PTI responses (Figure 1). Although a significant breakthrough has been achieved during the past decade, we are still far from full elucidation of the mechanisms underlying DAMP-regulated immune signaling. The following issues are still deserved to be figured out: (i) Identifying other plant endogenous molecules which function as DAMPs and the DAMP receptors; (ii) Deciphering mechanisms used by plants to transduce DAMP signaling and achieve local or systemic resistance. (iii) Integrating the DAMP signaling with other signaling pathways involved in plant resistance to pathogens, herbivores, or abiotic stresses. These studies will help us to better understand the plant immunity system and carry out the breeding of broad-spectrum and durable disease-resistant crops in the future.

## Author Contributions

SH wrote the manuscript. ZL, HS, and DW revised the manuscript.

## Conflict of Interest Statement

The authors declare that the research was conducted in the absence of any commercial or financial relationships that could be construed as a potential conflict of interest.
